# Comparative genomic and phenotypic analysis of *Escherichia coli* ST1193 and ST131 from urinary and bloodstream infections: insights into resistance, virulence, and divergent strategies

**DOI:** 10.1186/s12879-025-12044-5

**Published:** 2025-12-24

**Authors:** Qian Zeng, Su Wang, Shuzhen Xiao, Huifang Liu, Feifei Gu, Lizhong Han, Xiaofei Jiang

**Affiliations:** 1 Department of Laboratory Medicine, Huashan Hospital, Fudan University, Shanghai, China; 2 Department of Laboratory Medicine, Huadong Hospital, Fudan University, Shanghai, China; 3 Department of Laboratory Medicine, Ruijin Hospital, Shanghai Jiao Tong University School of Medicine, Shanghai, China; 4 Center for Infectious Diseases, Vision Medicals Co., Ltd, Guangzhou, Guangdong China

**Keywords:** ExPEC, ST1193, ST131, Antimicrobial resistance, Virulence, FimH

## Abstract

**Background:**

The global dissemination of multidrug-resistant (MDR) *Escherichia coli* (*E. coli*) clones, particularly the pandemic ST131 and the emerging ST1193, poses a serious public health threat. While ST131 is well-studied, the factors enabling ST1193’s rapid dissemination across both urinary and bloodstream infection niches remain unclear.

**Methods:**

We compared 48 urinary (UPEC) and bloodstream (BPEC) *E. coli* isolates of ST1193 (*n* = 24) and ST131 (*n* = 24) from Shanghai, using integrated phenotypic-genotypic analysis, including antimicrobial susceptibility, whole-genome sequencing (WGS), hemolysin assays, *Galleria mellonella* larval infection model.

**Results:**

Both lineages exhibited high rates of multidrug resistance with strongly genotype-phenotype concordance. Key contrasts emerged: ST1193-BPEC showed significantly higher resistance to trimethoprim/sulfamethoxazole than ST131-BPEC (*p* < 0.05), while ST131 overall demonstrated greater resistance to cefotaxime/cefazolin and a higher ESBL prevalence (ST131 83.33% vs. ST1193 50.00%, *p* < 0.05). All ST1193 isolates were resistant to fluoroquinolones but remained susceptible to carbapenems and ceftazidime-avibactam. Genomic analysis revealed striking conservation in ST1193, with near-uniform presence of *fimH*64 (23/24), K1 capsule (100%), and O75:H5 serotype (91.7%), along with unique virulence genes (*vat*, *neuABCD*, *hcp2*, *kpsT*, *espL1*). In contrast, ST131 displayed heterogeneity in *fimH* alleles (predominantly *fimH*30), K5 capsule (95.8%), and serotypes (O25:H4/O16:H5). Hemolysin production was exclusive to ST131 isolates (5/24). Virulence assays revealed divergent patterns: ST131-UPEC isolates showed significantly higher lethality in *G. mellonella* than ST131-BPEC (*p* = 0.024), whereas ST1193 virulence remained consistent across niches (UPEC vs. BPEC: *p* = 0.843).

**Conclusions:**

Our study confirms that ST1193 and ST131 are high-risk MDR clones employing distinct strategies—genomic conservation in ST1193 versus heterogeneity in ST131. Both exhibit substantial virulence and resistance, with *fimH*64 and *fimH*30 representing stable, lineage-specific markers. These conserved FimH variants present promising targets for future anti-adhesion therapeutics. Further mechanistic and multi-center studies are warranted to validate these findings.

**Supplementary Information:**

The online version contains supplementary material available at 10.1186/s12879-025-12044-5.

## Introduction

*Escherichia coli* (*E. coli*) is one of the most common clinical pathogens and is able to cause various infectious diseases both inside and outside of the intestinal tract [1]. Extraintestinal pathogenic *E. coli* (ExPEC) is a globally concerned pathogen and the main cause of urinary tract infections (UTIs) and bloodstream infections (BSIs) in adults, causing a variety of diseases affecting all ages [2, 3]. The treatment of these infections is increasingly challenged by antimicrobial resistance (AMR), recognized as a severe global public health concern that demands perspective combating strategies and the development of new talented antibiotics [4]. Currently, AMR poses an underrated threat to global public health [5]. Additionally, confronting the alarming increase in antibiotic resistance worldwide, *E. coli* is the most affected species within the *Enterobacterium* by extended-spectrum β-lactamases (ESBLs), with increasing prevalence of CTX-M-type ESBLs [6, 7].

ExPEC isolates are also notorious for harboring clinically significant antibiotic resistance genes, and the financial burden and clinical outcomes of ExPEC infections are evolving and challenging [8]. The complexity of UTIs is further underscored by diverse host-pathogen interactions, which influence disease outcomes and treatment efficacy [9]. Among these resistance mechanisms, the distribution of acquired carbapenemase genes among uropathogenic *E. coli* represents a particularly serious threat, rendering last-resort antibiotics ineffective [10]. Furthermore, the pathogenicity of ExPEC, including its ability to colonize and persist, is facilitated by various virulence factors, such as the distribution of chaperone-usher fimbriae and curli fimbriae among uropathogenic *E. coli* [11]. Interesting, the main reservoir of ExPEC is still the intestinal tract [12], which to some extent implies the potential of microbiome-targeted intervention.

Multilocus sequence typing (MLST) is a common method to assess genetic relatedness among strains, and *E. coli* exhibits high diversity in sequence types (STs) [13]. *E. coli* ST131 is the dominant lineage in global ExPEC isolates and one of the most successful multidrug-resistant (MDR) clones. Furthermore, ST131 is considered highly pathogenic due to its abundance of virulence-associated genes and association with diverse infections in healthcare and community settings [14]. Conversely, *E. coli* ST1193 is an emerging fluoroquinolone-resistant lineage, exhibiting a remarkable rise in BSIs and UTIs over the past decade [15, 16]. The virulence gene profiles in ST1193 suggest significant pathogenic potential [17]. In addition, the virulence in vivo of ExPEC is usually evaluated by the well-established *Galleria mellonella* larval infection model [18].

While the global dissemination of ST131 has been extensively documented [19, 20], with its epidemiological success attributed to a combination of phylogenetic background and specific phenotypic traits including virulence and antimicrobial resistance [21, 22], comparable studies on the emerging ST1193 clone remain limited. Given the convergent evolution of multidrug resistance in these two lineages, a comparative analysis of their genomic and phenotypic characteristics is urgently needed. This study aims to systematically compare clinical ST1193 and ST131 isolates from both urinary and bloodstream infections through integrated genomic and phenotypic analyses. Specifically, we seek to: (1) compare their antimicrobial resistance profiles and virulence gene repertoires; (2) assess their pathogenic potential using the *G. mellonella* infection model; and (3) identify lineage-specific molecular features that may contribute to their ecological adaptation and epidemic success. Our findings provide new insights into the comparative biology of these two high-risk clones and lay the groundwork for developing targeted interventions against emerging ExPEC pathogens.

## Materials and methods

### Study design and workflow

**Fig. 1:**
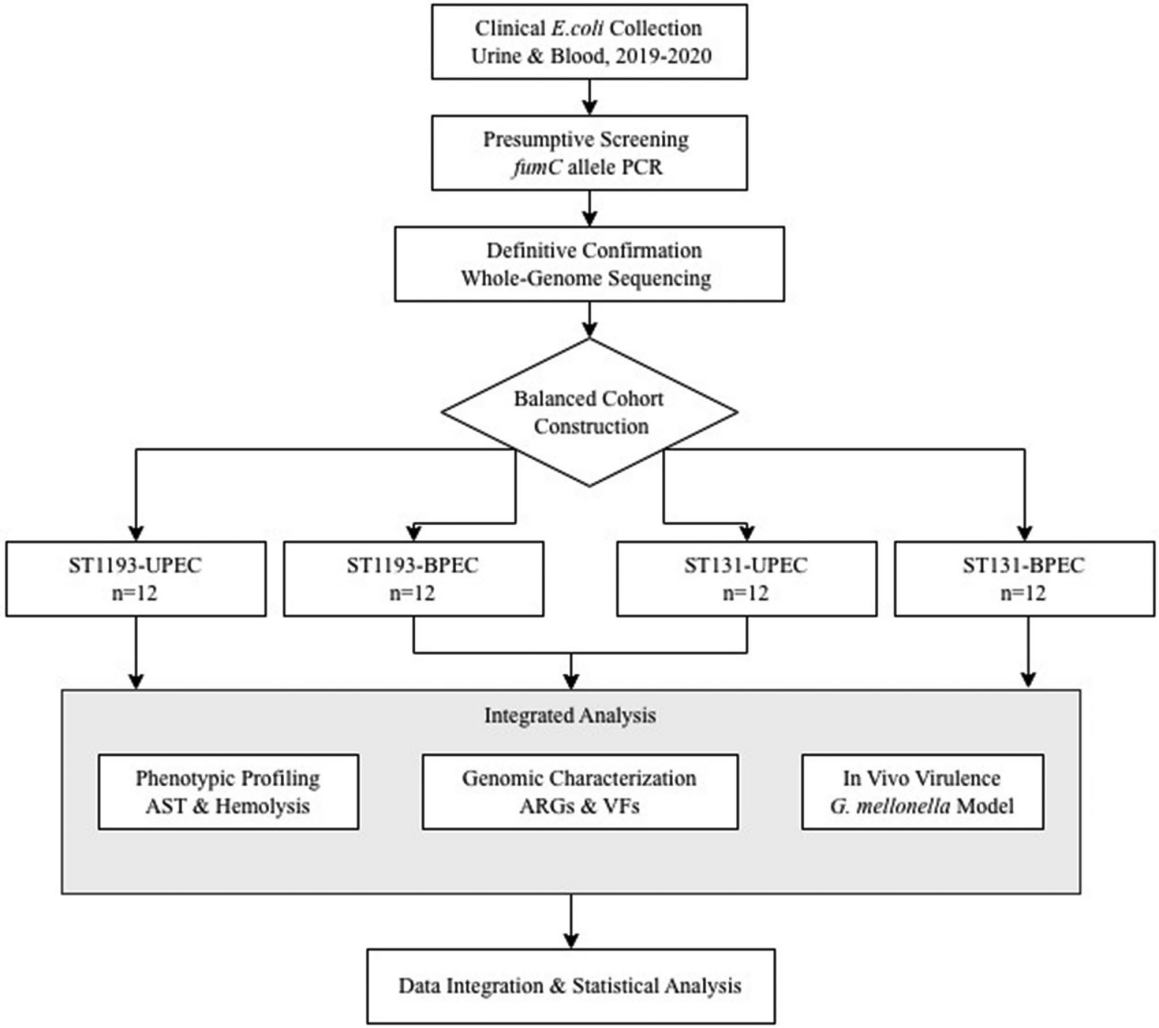
Schematic workflow of the study

A schematic overview of the experimental design is presented in Fig. 1. Briefly, a balanced cohort of *E. coli* clinical isolates was established based on sequence type (ST131 vs. ST1193) and infection site (urine vs. blood). These isolates were then subjected to an integrated analysis encompassing whole-genome sequencing for genomic characterization, a series of phenotypic assays, and in vivo virulence assessment, as detailed in the following sections.

A balanced cohort of 48 *E. coli* isolates (12 per group) comprising sequence types ST131 and ST1193 from urinary and bloodstream infections was established. All isolates underwent comprehensive genomic characterization (antibiotic resistance and virulence genotyping, plasmid analysis, and phylogenomic), phenotypic profiling (antimicrobial susceptibility testing and hemolysis assay), and in vivo virulence assessment using the *G. mellonella* infection model. The results were integrated through statistical analysis for comparative biology insights.

### Bacterial collection

From July 2019 to June 2020, *E. coli* strains were collected from mid-stream urine samples and blood culture specimens at Ruijing Hospital in Shanghai. Initial presumptive identification of ST131 and ST1193 clones from the collection was performed by screening for specific *fumC* alleles, a method established in the literature [23]. Subsequently, whole-genome sequencing (WGS) was used for definitive confirmation of sequence types.

To construct a balanced cohort for comparative analysis, isolates were strategically selected. From the bloodstream infection (BSI) collection, all available ST131 (*n* = 12) and ST1193 (*n* = 12) isolates were included. From the much larger urinary tract infection (UTI) collection, a random selection (using Excel’s random number generator) was performed to ensure temporal representation and to yield an equal number of isolates, resulting in 12 ST131 UPEC and 12 ST1193 UPEC isolates. As it is limited by the quantity of ST1193 from bloodstream infections, the selection process resulted in a final cohort of 48 isolates, comprising four balanced groups of 12 isolates each: (1) ST1193 from blood (BPEC), (2) ST1193 from urine (UPEC), (3) ST131 from blood (BPEC), and (4) ST131 from urine (UPEC). The distribution of all identified *fumC* types in the original collections is provided in Supplementary Table S1.

### Multilocus sequencing typing

MLST was performed based on seven conserved housekeeping genes (*adk*, *fumC*, *gyrB*, *icd*, *mdh*, *purA*, and *recA*). Following whole-genome sequencing, sequence types (STs) were determined according to allelic profiles of these loci (https://bigsdb.pasteur.fr/klebsiella/). Only isolates identified as ST131 or ST1193 were enrolled in this study.

### Antimicrobial susceptibility test

Antimicrobial susceptibility was assessed using the disk diffusion method, with concurrent detection of ESBL-producing isolates. Results were interpreted per the Clinical and Laboratory Standards Institute (CLSI) 2021 guidelines [24]. Notably, fosfomycin and nitrofurantoin interpretations applied 2021 CLSI UTI-specific breakpoints, while tigecycline susceptibility was evaluated according to European Committee on Antimicrobial Susceptibility Testing (EUCAST) standards [25].

The tested antibiotics (with disk potencies) were categorized as follows: Penicillin/β-lactamase inhibitors [piperacillin/tazobactam (TZP, 100/10 µg)]; Cephalosporins [cefazolin (KZ, 30 µg), cefotaxime (CTX, 30 µg), ceftazidime (CAZ, 30 µg)]; Carbapenems [imipenem (IMP, 10 µg), meropenem (MEM, 10 µg)]; Monobactams [aztreonam (ATM, 30 µg)]; Aminoglycosides [gentamicin (GEN, 10 µg), tobramycin (TOB, 10 µg), amikacin (AK, 30 µg)]; Fluoroquinolones [ciprofloxacin (CIP, 5 µg), levofloxacin (LEV, 5 µg)]; Tetracyclines/Glycylcyclines [doxycycline (DOX, 30 µg), minocycline (MI, 30 µg), tigecycline (TIG, 15 µg)]; Folate pathway antagonists [trimethoprim/sulfamethoxazole (SXT, 1.25/23.75 µg)]; Nitrofurans [nitrofurantoin (AHD, 300 µg)]; Epoxides [fosfomycin (FOS, 200 µg)]; β-lactam/β-lactamase inhibitor combinations for ESBL confirmation [ceftazidime/clavulanate (CCV, 30/10 µg), cefotaxime/clavulanate (CTC, 30/10 µg)]; and Novel β-lactam/β-lactamase inhibitor combinations [ceftazidime/avibactam (CZA, 30/20 µg)]. Quality control was performed using: *E. coli* ATCC35218, *Pseudomonas aeruginosa* ATCC27853, *Klebsiella pneumoniae* ATCC700603 and *E.coli* ATCC25922.

Multidrug resistance (MDR) was defined as non-susceptibility to at least one agent in three or more antimicrobial categories [26, 27]. Furthermore, the definitions for extensively drug-resistant (XDR) and pandrug-resistant (PDR) were applied in accordance with international standardized criteria [28]. Specifically, XDR was defined as non-susceptibility to at least one agent in all but two or fewer antimicrobial categories (i.e., bacterial isolates remain susceptible to only one or two categories). PDR was defined as non-susceptibility to all agents in all antimicrobial categories tested.

### DNA sequencing and analysis

Strains isolated from single colonies were first grown overnight in LB broth. Subsequently, genomic DNA was extracted from each isolate using the Bacterial Genome DNA Extraction Kit (TIANGEN). Immediately following extraction, DNA concentration was quantified with a Qubit Fluorometer 3.0. For library preparation, the TIANSeq Direct Rapid DNA Library Construction Kit (Illumina platform) was employed. To ensure library quality, both concentration and fragment length distribution were assessed using the Qubit Fluorometer 3.0 and Agilent 2100 Bioanalyzer, respectively. Based on the calculated effective concentrations and target sequencing depth requirements, qualified libraries were then pooled in equimolar ratios. This pooled library was subjected to paired-end sequencing (PE150) on the Illumina NovaSeq platform to generate 150-bp raw reads. Finally for data processing, raw sequencing reads underwent quality control through fastp (https://github.com/OpenGene/fastp), which performed adapter trimming and filtered low-quality reads.

Next, clean reads were firstly assembled using MEGAHIT [29]. Subsequently, genome assembly quality was evaluated with QUAST software [30]. Following assembly, Prokka was employed for comprehensive genome annotations [31]. To characterize functional genetic elements, gene content analysis was performed using specialized tools: Specifically, antibiotic resistance genes and mutations were identified using the Comprehensive Antibiotic Resistance Database (CARD) [32], with perfect/strict match criteria applied to ensure stringent selection. For virulence profiling, the core dataset (setA) of Virulence Factors Database (VFDB) was interrogated via DIAMOND [33, 34], implementing cutoff thresholds of 1 × 10^− 30^ and 90% identity based on protein alignment. Regarding mobile genetic elements, plasmid replicon types (including IncF subtypes) were determined by PlasmidFinder and pMLST [35], using 95% minimum identity and 60% minimum coverage thresholds. Concurrently, serotyping was performed via SerotypeFinder (85% identity, 60% coverage), while *fimH* alleles were characterized using FimTyper (95% identity). Finally, capsular typing (K1/K5) was conducted through protein sequence alignment against KpsM/D/E/T genes (groups 1 and 5), where 100% identity matches were required to assign capsule types [36]. The target genes analyzed in this study, encompassing antibiotic resistance genes and key virulence factors, along with their characteristics and analytical approaches, are summarized in Table 1.

**Table 1:** Characteristics of target genes analyzed in this study

Category	Gene(s) / Locus	Primary Function / Phenotypic Association	Detection Method / Criteria
Antibiotic Resistance	*gyrA* (S83L, D87N), *parC* (S80I, E84V)	Fluoroquinolone resistance	CARD / Point mutation screening
	*bla*_CTX−M_ variants	ESBL production, 3rd-gen cephalosporin resistance	CARD
	*aac(3)-IId*, *aph(3’’)-Ib*,* aph(6)-Id*,* rmtB*	Pan-aminoglycoside resistance (16S rRNA methyltransferase)	CARD
	*tet(A)*, *tet(B)*	Tetracycline resistance	CARD
	*sul1*,* sul2*	Sulfonamide resistance	CARD
	*dfrA17*	Trimethoprim resistance	CARD
Virulence Factors	*fimH* alleles (*fimH*30, *fimH*41, *fimH*64)	Adhesion, tissue tropism	FimTyper (95% identity)
	*hlyCABD*	Hemolysin production, cytotoxicity	VFDB (90% identity, 1e-30 e-value)
	*cnf1*	Cytotoxic necrotizing factor	VFDB (90% identity, 1e-30 e-value)
	P fimbriae operon (*papA*/*C*/*G*)	Adhesion to urinary tract	VFDB (90% identity, 1e-30 e-value)
	*vat*	Vacuolating autotransporter toxin	VFDB (90% identity, 1e-30 e-value)
	*neuABCD*, *kpsMTED*	K1 capsule synthesis, serum resistance	VFDB & Protein Alignment (100% identity to KpsM/D/E/T)
	*hcp2*	Type VI secretion system (T6SS) structural component	VFDB (90% identity, 1e-30 e-value)
	*espL1*	Type III secretion system effector	VFDB (90% identity, 1e-30 e-value)
	*clbA-clbS*	Genotoxin synthesis	VFDB (90% identity, 1e-30 e-value)
	*Afa/Dr* adhesins	Diffusely adhering adhesins	VFDB (90% identity, 1e-30 e-value)
	*pic*	Serine protease autotransporter	VFDB (90% identity, 1e-30 e-value)
Strain Typing	*fumC* allele	Presumptive screening marker for ST131/ST1193	PCR & WGS
	Serotype (O and H antigens)	Serological typing (e.g., O75:H5, O25:H4)	SerotypeFinder (85% identity, 60% coverage)
	Plasmid Replicons (e.g., IncFIA, IncFIB, IncFII)	Plasmid epidemiology and classification	PlasmidFinder (95% identity, 60% coverage)

### Phylogenetic analysis

OrthoFinder was employed to identify single-copy orthologs across all strains [37]. Subsequently, protein-coding sequences of these orthologous genes underwent multiple sequence alignment using MUSCLE under default parameters, and conserved sites were extracted with Gblocks using its default settings [38, 39]. Based on this curated dataset, a maximum-likelihood phylogenomic tree was constructed with PhyML, incorporating 100 bootstrap replicates for node support estimation [40]. Finally, the phylogenetic tree with accessory traits was visualized via the Interactive Tree of Life (iTOL) platform [41], while virulence gene distribution patterns were illustrated through a heatmap generated in RStudio.

### Hemolysis assays

Overnight bacterial cultures were washed with phosphate-buffered saline (PBS) and resuspended in PBS. Subsequently, hemolysis assays were performed by inoculating 5 µL aliquots of each isolate onto Columbia blood agar plates, followed by overnight incubation at 37 °C [42].

### *G. mellonella* larvae infection model

The virulence of all 48 isolates in the study cohort was assessed using the *G. mellonella* larvae infection model to ensure a comprehensive and unbiased comparison across the four predefined groups (ST1193-UPEC, ST1193-BPEC, ST131-UPEC, ST131-BPEC).

The *G. mellonella* larvae infection was performed as previously described with modifications [43, 44]. *G. mellonella* larvae were maintained in darkness at room temperature and utilized within seven days of receipt. Healthy larvae exhibiting cream coloration and motility were selected based on uniform size (∼3 cm length) and weight (500–600 mg). Bacterial strains were cultured overnight, washed three times with phosphate-buffered saline (PBS), and resuspended in PBS. The inoculum concentration was standardized to 3 × 10⁸ CFU/mL, verified via colony counting. A 10-µL aliquot of this suspension was injected into each larva, with ten larvae inoculated per ExPEC strain. Through the entire experiments, the PBS was an inoculation control and hypervirulent *K. pneumoniae* RJF293 was a positive control [45, 46]. Inoculated larvae were incubated at 37 °C in darkness for 96 h. Survival and phenotypic responses (activity, cocoon formation, melanization) were scored at 24-hour intervals using the criteria in Figure S1 and Table S2. Larvae showing no body movement or leg reflex were considered dead and removed. All infection experiments (48 strains) were performed in triplicate. Survival curves were generated using GraphPad Prism 9, while virulence scores were presented as histograms with normal distribution curves.

### Statistical analysis

Statistical analyses were performed using SPSS Statistics 26 (IBM, Armonk, NY). Categorical variables were compared between groups using the Chi-square test, Fisher’s exact test, or continuity correction, as appropriate. *G. mellonella* virulence scores across the four experimental groups (ST1193-UPEC, ST1193-BPEC, ST131-UPEC, ST131-BPEC) were analyzed via the Kruskal-Wallis test, and the Mann-Whitney U test was used for pairwise comparisons between the four groups. A two-tailed *p*-value threshold of 0.05 defined statistical significance.

## Results

### Antimicrobial susceptibility and antibiotic resistance genes

All 48 isolates remained susceptible to carbapenems (meropenem, imipenem) and the novel β-lactam/β-lactamase inhibitor combination ceftazidime/avibactam. Direct comparison within each infection source revealed few differences, with ST1193 BPEC isolates exhibiting significantly higher resistance to trimethoprim/sulfamethoxazole than their ST131 counterparts (*p* < 0.05).

In contrast, pooled analysis across sources uncovered distinct, lineage-specific resistance profiles. ST131 isolates showed significantly higher resistance rates to cefotaxime and cefazolin, whereas ST1193 isolates were significantly more resistant to trimethoprim/sulfamethoxazole, ciprofloxacin, and levofloxacin. This finding underscores the convergent evolution of multidrug resistance in these two successful lineages, albeit through different genetic determinants. More detailed drug resistance results are presented in Table 2. Notably, universal resistance to fluoroquinolones in all 24 ST1193 isolates was concordant with their universal carriage of the characteristic *gyrA* (S83L, D87N) and *parC* (S80I, E84V) allele combinations, a hallmark of this clone.

The prevalence of ESBL production was significantly higher in ST131 (83.3%; 20/24) than in ST1193 (50%; 12/24) (*p* < 0.05). A more nuanced, stratified analysis revealed two key epidemiological patterns: Firstly, ESBL producers were more prevalent among bloodstream isolates (BSI) than urinary isolates (UTI) within both STs (ST1193-BSI: 8/12 vs. ST1193-UTI: 4/12; ST131-BSI: 12/12 vs. ST131-UTI: 8/12). Secondly, ST131 demonstrated a higher prevalence of ESBL production than ST1193, regardless of source. Genotypic analysis confirmed that resistance to third-generation cephalosporins was primarily mediated by *bla*_CTX−M−_ variants. Among these, *bla*_CTX−M−27_ was unexpectedly the most prevalent in both ST131 and ST1193 isolates, surpassing *bla*_CTX−M−15_ in ST131. The higher ESBL burden in ST131, particularly among invasive isolates, may contribute to its persistence in healthcare settings.

Based on standardized international definitions, our analysis revealed a high and comparable prevalence of multidrug-resistant (MDR) phenotypes in both lineages (70.8% in ST131 vs. 75% in ST1193; *p* > 0.05). A subset of these MDR isolates met the more stringent criteria for extensively drug-resistant (XDR). Although the proportion of XDR isolates was higher in ST1193 (16.7%, 4/24) than in ST131 (4.2%, 1/24), this difference did not reach statistical significance (*p* = 0.35, Fisher’s exact test). Importantly, no pandrug-resistant (PDR) strains were identified, which is consistent with the universal susceptibility to key last-resort antibiotics. Additionally, one ST131 isolate harboring *rmtB* exhibited pan-aminoglycoside resistance. Complete resistance profiles and genotypic correlations are detailed in Tables 2 and 3, respectively. All ESBL strains have been marked gray and are displayed in Table 3.

**Table 2:** Antibiotic resistance rates of *E. coli* ST131 and ST1193 strain

Agent	UPEC		BPEC		ST1193	ST131
	ST1193 (*n* = 12)	ST131(*n* = 12)	ST1193 (*n* = 12)	ST131 (*n* = 12)	(*n* = 24)	(*n* = 24)
Ceftazidime	16.67%	8.33%	33.33%	75.00%	25.00%	41.67%
Cefotaxime	33.33%	66.67%	66.67%	100.00%	**50.00%**	**83.33%**
Cefazolin	33.33%	66.67%	66.67%	100.00%	**50.00%**	**83.33%**
Piperacillin/tazobactam	0.00%	0.00%	0.00%	0.00%	0.00%	0.00%
Ceftazidime/avibactam	0.00%	0.00%	0.00%	0.00%	0.00%	0.00%
Ciprofloxacin	100.00%	66.67%	100.00%	83.33%	100.00%	75.00%
Levofloxacin	100.00%	66.67%	100.00%	83.33%	100.00%	75.00%
Gentamicin	33.33%	25.00%	50.00%	33.33%	41.67%	29.17%
Amikacin	0.00%	0.00%	0.00%	8.33%	0.00%	4.17%
Tobramycin	25.00%	16.67%	25.00%	33.33%	25.00%	25.00%
Meropenem	0.00%	0.00%	0.00%	0.00%	0.00%	0.00%
Imipenem	0.00%	0.00%	0.00%	0.00%	0.00%	0.00%
Doxycycline	33.33%	25.00%	8.33%	8.33%	20.83%	16.67%
Minocycline	8.33%	0.00%	8.33%	0.00%	8.33%	0.00%
Tigecycline	0.00%	0.00%	8.33%	0.00%	4.17%	0.00%
Aztreonam	16.67%	41.67%	16.67%	41.67%	16.67%	41.67%
Fosfomycin-UTI	0.00%	0.00%	/	/	/	/
Nitrofurantoin-UTI	0.00%	0.00%	/	/	/	/
Trimethoprim/sulfamethoxazole	58.33%	50.00%	83.33%	25.00%	70.83%	37.50%

The yellow areas/bold number indicate statistically significant differences (*p* < 0.05) in different pairwise comparisons (ST1193 of UPEC vs. ST131 of UPEC, ST1193 of BPEC vs. ST131 of BPEC, ST1193 vs. ST131)

**Table 3:** Antibiotic resistance genes and antibiotic resistance profiles of expec isolates

Group	Isolate ID	*bla* gene	QNR	AME/16S rRNA methylases	Other antibiotic resistance genes	Antibiotic resistance profiles
ST1193 of UPEC	ExPEC5	*bla*_TEM−1_	*gyrA*: D87N, S83L, *parC*: S80I	*aac(3)-IId*	/	CIP-LEV-GEN-TOB
	ExPEC137	*bla*_TEM−1_	*gyrA*: D87N, S83L, *parC*: S80I	/	*tet(B)*, *tetR*, *dfrA17*	SXT-CIP-LEV-DOX
	**ExPEC172**	*bla*_TEM−1_, *bla*_CTX−M−64_	*gyrA*: D87N, S83L, *parC*: S80I	*aac(3)-IId*, *aph(3’’)-Ib*, *aph(6)-Id*	*tet(A)*, *sul1*, *sul2*, *dfrA17*	ATM-SXT-CAZ-CTX-KZ-CIP-LEV-GEN-TOB
	ExPEC425	*bla*_TEM−1_	*gyrA*: D87N, S83L, *parC*: S80I	/	/	CIP-LEV
	**ExPEC529**	*bla*_TEM−1_, *bla*_CTX−M−14_	*gyrA*: D87N, S83L, *parC*: S80I	/	/	atm-CTX-KZ-CIP-LEV
	**ExPEC609**	bla_TEM−1_, *bla*_CTX−M−55_	*gyrA*: D87N, S83L, *parC*: S80I	*aph(6)-Id*	*tet(B)*, *tetR*, *sul2*, *dfrA17*	ATM-SXT-CAZ-CTX-KZ-CIP-LEV-DOX
	ExPEC863	*bla*_TEM−1_	*gyrA*: D87N, S83L, *parC*: S80I	*aac(3)-IId*	*sul1*,* dfrA17*	SXT-CIP-LEV-GEN-TOB
	ExPEC999	*bla*_TEM−1_	*gyrA*: D87N, S83L, *parC*: S80I	/	/	CIP-LEV
	**ExPEC1275**	*bla*_CTX−M−27_	*gyrA*: D87N, S83L, *parC*: S80I	/	*tet(B)*, *tetR*	SXT-CTX-KZ-CIP-LEV-DOX-mi
	ExPEC1341	*bla*_TEM−1_	*gyrA*: D87N, S83L, *parC*: S80I	*aph(3’’)-Ib*, *aph(6)-Id*	*tet(A)*, *sul2*	sxt-CIP-LEV-DOX-MI
	ExPEC1465	*bla*_TEM−1_	*gyrA*: D87N, S83L, *parC*: S80I	*aac(3)-IId*	*sul1*, *dfrA17*	SXT-CIP-LEV-GEN-tob
	ExPEC1600	*bla*_TEM−1_	*gyrA*: D87N, S83L, *parC*: S80I	*aph(3’’)-Ib*, *aph(6)-Id*	*tet(A)*, *sul1*, *sul2*, *dfrA17*	SXT-CIP-LEV
ST1193 of BPEC	**ExPEC2201**	*bla*_TEM−1_, *bla*_CTX−M−64_	*gyrA*: D87N, S83L, *parC*: S80I	*aac(3)-IId*, *aph(3’’)-Ib*, *aph(6)-Id*	*tet(A)*, *sul1*, *sul2*, *dfrA17*	ATM-SXT-CAZ-CTX-KZ-CIP-LEV-GEN-tob-dox
	**ExPEC2214**	*bla*_CTX−M−27_	*gyrA*: D87N, S83L, *parC*: S80I	*aph(3’’)-Ib*, *aph(6)-Id*	*tet(A)*, *sul1*, *sul2*, *dfrA17*	SXT-caz-CTX-KZ-CIP-LEV
	**ExPEC2215**	*bla*_CTX−M−27_	*gyrA*: D87N, S83L, *parC*: S80I	*aph(3’’)-Ib*, *aph(6)-Id*	*tet(A)*, *sul1*, *sul2*, *dfrA17*	atm-SXT-CAZ-CTX-KZ-CIP-LEV
	**ExPEC2252**	*bla*_CTX−M−27_	*gyrA*: D87N, S83L, *parC*: S80I	*aph(3’’)-Ib*, *aph(6)-Id*	*tet(A)*, *sul1*, *sul2*, *dfrA17*	SXT-caz-CTX-KZ-CIP-LEV
	ExPEC2292	/	*gyrA*: D87N, S83L, *parC*: S80I	/	/	CIP-LEV
	ExPEC2349	*bla*_TEM−1_	*gyrA*: D87N, S83L, *parC*: S80I	*aac(3)-IId*, *aph(3’’)-Ib*, *aph(6)-Id*	*tet(A)*, *sul1*, *sul2*, *dfrA17*	SXT-CIP-LEV-GEN-tob
	**ExPEC2419**	*bla*_CTX−M−27_	*gyrA*: D87N, S83L, *parC*: S80I	*aph(3’’)-Ib*, *aph(6)-Id*	*tet(A)*, *sul1*, *sul2*, *dfrA17*	SXT-CAZ-CTX-KZ-CIP-LEV
	ExPEC2580	*bla*_TEM−1_	*gyrA*: D87N, S83L, *parC*: S80I	*aac(3)-IId*	/	CIP-LEV-GEN-tob
	**ExPEC2650**	/	*gyrA*: D87N, S83L, *parC*: S80I	*aph(3’’)-Ib*, *aph(6)-Id*	*tet(A)*, *sul1*, *sul2*, *dfrA17*	ATM-SXT-CAZ-CTX-KZ-CIP-LEV-DOX-MI-TIG
	**ExPEC2757**	*bla*_TEM−1_, *bla*_CTX−M−14_	*aac(6’)-Ib-cr6*, *gyrA*: D87N, S83L, *parC*: S80I	*aac(3)-IId*	*sul1*, *dfrA17*, *dfrA27*	SXT-CTX-KZ-CIP-LEV-GEN-TOB
	ExPEC2987	*bla*_TEM−1_	*gyrA*: D87N, S83L, *parC*: S80I	*aac(3)-IId*, *aph(3’’)-Ib*, *aph(6)-Id*	*tet(A)*, *sul1*, *sul2*, *dfrA17*	SXT-CIP-LEV-GEN-TOB-dox
	**ExPEC3010**	*bla*_TEM−1_, *bla*_CTX−M−27_	*qepA2*, *gyrA*: D87N, S83L, *parC*: S80I	*aac(3)-IId*	*sul1*, *dfrA17*	SXT-caz-CTX-KZ-CIP-LEV-GEN-TOB
ST131 of UPEC	**ExPEC111**	*bla*_CTX−M−15_, *bla*_OXA−1_	*aac(6’)-Ib-cr5*, *gyrA*: D87N, S83L, *parC*: S80I	*aac(3)-IIe*	*tet(A)*, *sul1*, *dfrA17*	ATM-SXT-caz-CTX-KZ-CIP-LEV-GEN-TOB
	**ExPEC658**	*bla*_CTX−M−27_	*gyrA*: D87N, S83L, *parC*: S80I	/	/	ATM-CTX-KZ-cip-lev-TOB
	ExPEC761	/	*gyrA*: D87N, S83L, *parC*: S80I	/	/	CIP-LEV
	**ExPEC782**	*bla*_TEM−1_, *bla*_CTX−M−55_	*gyrA*: S83L	*aac(3)-IId*	/	ATM-CAZ-CTX-KZ-CIP-LEV-GEN-tob
	**ExPEC904**	*bla*_TEM−1_, *bla*_CTX−M−14_	*gyrA*: D87N, S83L, *parC*: S80I	/	/	ATM-CTX-KZ-CIP-LEV-tzp
	**ExPEC1053**	*bla*_CTX−M−14_	*gyrA*: D87N, S83L, *parC*: S80I	/	/	CTX-KZ-CIP-LEV
	ExPEC1144	*bla*_TEM−1_	*gyrA*: D87Y, S83L, *parC*: S80I	/	*dfrA17*	SXT-CIP-LEV
	ExPEC1167	*bla*_TEM−1_	*gyrA*: S83L, *parC*: S80I	*aac(3)-IId*, *aph(3’’)-Ib*, *aph(6)-Id*	*tet(A)*, *sul1*, *sul2*, *dfrA17*	SXT-GEN-tob-DOX
	**ExPEC1213**	*bla*_CTX−M−27_	*gyrA*: D87N, S83L, *parC*: S80I	*aph(3’’)-Ib*, *aph(6)-Id*	*tet(A)*, *sul1*, *sul2*, *dfrA17*	ATM-SXT-CTX-KZ-CIP-LEV-DOX
	**ExPEC1254**	*bla*_CTX−M−14_	*gyrA*: D87N, S83L, *parC*: S80I	*aph(3’’)-Ib*, *aph(6)-Id*	*tet(A)*, *sul1*, *sul2*, *dfrA17*	atm-SXT-CTX-KZ-CIP-LEV-DOX
	ExPEC1265	*bla*_TEM−1_	*gyrA*: S83L	/	/	lev
	**ExPEC1284**	*bla*_CTX−M−15_	*gyrA*: S83L	/	*sul1*, *dfrA17*	atm-SXT-CTX-KZ
ST131 of BPEC	**ExPEC2220**	*bla*_CTX−M−27_	*gyrA*: D87N, S83L, *parC*: S80I	*aph(3’’)-Ib*, *aph(6)-Id*	*tet(A)*, *sul2*	atm-CAZ-CTX-KZ-CIP-LEV-DOX
	**ExPEC2299**	*bla*_TEM−1_, *bla*_CTX−M−15_, *bla*_OXA−1_	*gyrA*: D87N, S83L, *parC*: S80I	*aac(3)-IId*, *aph(3’’)-Ib*, *aph(6)-Id*	*tet(A)*, *sul1*, *sul2*, *dfrA17*	ATM-SXT-CAZ-CTX-KZ-CIP-LEV-GEN-TOB-dox
	**ExPEC2434**	*bla*_CTX−M−14_	*gyrA*: D87N, S83L, *parC*: S80I	/	/	atm-CTX-KZ-CIP-LEV
	**ExPEC2459**	*bla*_TEM−1_, *bla*_CTX−M−27_	*gyrA*: S83L	*aph(3’’)-Ib*, *aph(6)-Id*	*tet(A)*, *sul1*, *sul2*, *dfrA17*	SXT-CAZ-CTX-KZ
	**ExPEC2461**	*bla*_CTX−M−27_	*gyrA*: D87N, S83L, *parC*: S80I	/	/	caz-CTX-KZ-CIP-LEV
	**ExPEC2503**	*bla*_CTX−M−15_, *bla*_OXA−1_	*aac(6’)-Ib-cr6*, *gyrA*: D87N, S83L, *parC*: S80I	/	/	ATM-CAZ-CTX-KZ-CIP-LEV-TOB
	**ExPEC2583**	*bla*_TEM−1_, *bla*_CTX−M−27_	*gyrA*: S83L	/	/	CAZ-CTX-KZ
	**ExPEC2592**	*bla*_TEM−1_, *bla*_CTX−M−14_	*gyrA*: D87N, S83L, *parC*: S80I	*aac(3)-IIc*, *rmtB*	*tet(A)*	CTX-KZ-CIP-LEV-GEN-AK-TOB-dox
	**ExPEC2925**	*bla*_CTX−M−27_	*gyrA*: D87N, S83L, *parC*: S80I	*aph(3’’)-Ib*, *aph(6)-Id*	*tet(A)*, *sul1*, *sul2*, *dfrA17*	SXT-CAZ-CTX-KZ-CIP-LEV-dox
	**ExPEC2946**	*bla*_CTX−M−27_	*gyrA*: D87N, S83L, *parC*: S80I	/	/	ATM-CAZ-CTX-KZ-CIP-LEV
	**ExPEC2960**	*bla*_TEM−1_, *bla*_CTX−M−15_	*gyrA*: D87N, S83L, *parC*: S80I	*aac(3)-IId*	/	ATM-CAZ-CTX-KZ-CIP-LEV-GEN-tob
	**ExPEC3011**	*bla*_CTX−M−15_, *bla*_OXA−1_	*aac(6’)-Ib-cr5*, *gyrA*: D87N, S83L, *parC*: S80I	*aac(3)-IIe*	*tet(A)*	ATM-CAZ-CTX-KZ-CIP-LEV-GEN-TOB-dox

The upper case of an antibiotic indicates that the strain is resistant to the antibiotic, and the lower case indicates that the strain is intermediate resistant to the antibiotic. ESBL-producing isolates are highlighted in capital letters

### Phylogeny, *fimH* alleles, capsular types, serotypes and plasmid replicons

Phylogenetic analysis confirmed the clear separation of ST1193 and ST131 into two distinct clades. Beyond this expected divergence, we focused on features that could inform their respective ecological adaptations.

A striking contrast was observed in the conservation of key typing markers. ST1193 exhibited near-complete homogeneity: 23/24 isolates carried the *fimH*64 allele (ExPEC999 harbored a novel allele), all produced the K1 capsule, and 22/24 belonged to serotype O75:H5. This remarkable consistency suggests a recent clonal expansion with minimal diversification. In contrast, ST131 displayed expected heterogeneity, segregating into its classic subclones: the H30 subclone (*n* = 16, serotype O25:H4, carrying *fimH*30) and the H41 subclone (*n* = 8, serotype O16:H5, carrying *fimH*41).

**Fig. 2:**
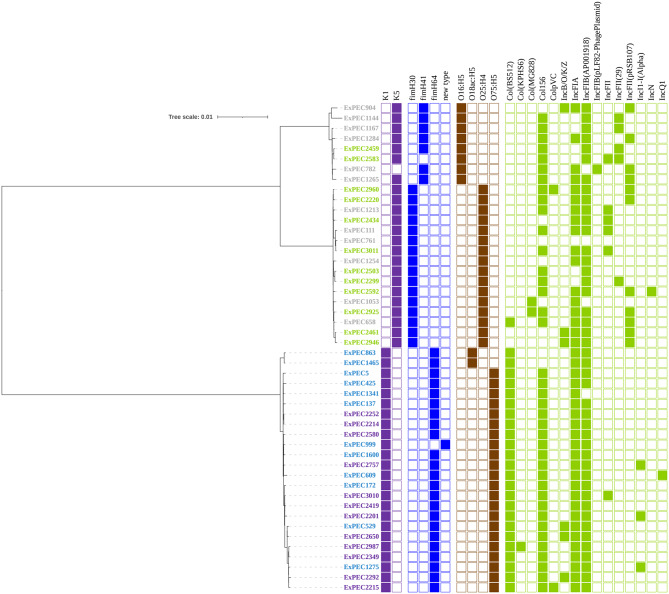
Phylogenetic tree with accessory features in 48 ExPEC isolates. In the colored strains, gray is ST131 of UPEC, bright green is ST131 of BPEC, blue is ST1193 of UPEC and purple is ST1193 of BPEC. Squares colored in different color represented the corresponding features

Plasmid replicon analysis also revealed lineage preferences. PlasmidFinder was used to detect the replicator types of plasmids, and IncF replicon types were identified in PubMLST database (Fig. 2; Table 4). The commonest replicon type in ST1193 isolates was the IncF group of IncF-FIA-FIB with the FAB formula F-:A1:B10 (54.17%), followed by FAB formula F-:A1:B1 (25%). Only one ST1193 isolate possessed IncFII. However, 87.5% (21/24) ST131 isolates possessed IncFII with the dominant IncFII-FIA-FIB complex exhibiting FAB formula F1:A2:B20 (37.50%). These distinct replicon profiles suggest differences in the underlying plasmid-mediated accessory gene acquisition and maintenance between the two lineages. All the relevant information is presented in Fig. 2; Table 4.

**Table 4:** Genomic characteristics and IncF plasmids of 48 isolates

Group	Isolate ID	capsule	serotype	*fimH*	CTX-M type	plasmid IncF system			pMLST
						FII	FIA	FIB	
ST1193 of UPEC	ExPEC5	K1	O75:H5	*fimH*64			FIA_1	FIB_20	F-:A1:B20
	ExPEC137	K1	O75:H5	*fimH*64			FIA_1	FIB_10	F-:A1:B10
	ExPEC172	K1	O75:H5	*fimH*64	CTX-M-64		FIA_1	FIB_10	F-:A1:B10
	ExPEC425	K1	O75:H5	*fimH*64			FIA_1	FIB_1	F-:A1:B1
	ExPEC529	K1	O75:H5	*fimH*64	CTX-M-14		FIA_1	FIB_10	F-:A1:B10
	ExPEC609	K1	O75:H5	*fimH*64	CTX-M-55		FIA_1	FIB_10	F-:A1:B10
	ExPEC863	K1	O18ac: H5	*fimH*64			FIA_1	FIB_1	F-:A1:B1
	ExPEC999	K1	O75:H5	new type			FIA_1	FIB_1	F-:A1:B1
	ExPEC1275	K1	O75:H5	*fimH*64	CTX-M-27		FIA_1	FIB_10	F-:A1:B10
	ExPEC1341	K1	O75:H5	*fimH*64			FIA_1		F-:A1:B-
	ExPEC1465	K1	O18ac: H5	*fimH*64			FIA_1	FIB_1	F-:A1:B1
	ExPEC1600	K1	O75:H5	*fimH*64			FIA_1	FIB_1	F-:A1:B1
ST1193 of BPEC	ExPEC2201	K1	O75:H5	*fimH*64	CTX-M-64		FIA_1	FIB_10	F-:A1:B10
	ExPEC2214	K1	O75:H5	*fimH*64	CTX-M-27		FIA_1	FIB_10	F-:A1:B10
	ExPEC2215	K1	O75:H5	*fimH*64	CTX-M-27		FIA_1	FIB_10	F-:A1:B10
	ExPEC2252	K1	O75:H5	*fimH*64	CTX-M-27		FIA_1	FIB_10	F-:A1:B10
	ExPEC2292	K1	O75:H5	*fimH*64			FIA_1	FIB_10	F-:A1:B10
	ExPEC2349	K1	O75:H5	*fimH*64			FIA_1	FIB_20	F-:A1:B20
	ExPEC2419	K1	O75:H5	*fimH*64	CTX-M-27		FIA_1	FIB_10	F-:A1:B10
	ExPEC2580	K1	O75:H5	*fimH*64			FIA_1	FIB_1	F-:A1:B1
	ExPEC2650	K1	O75:H5	*fimH*64			FIA_1	FIB_10	F-:A1:B10
	ExPEC2757	K1	O75:H5	*fimH*64	CTX-M-14		FIA_1	FIB_20	F-:A1:B20
	ExPEC2987	K1	O75:H5	*fimH*64			FIA_1	FIB_20	F-:A1:B20
	ExPEC3010	K1	O75:H5	*fimH*64	CTX-M-27	FII_2	FIA_1	FIB_10	F2:A1:B10
ST131 of UPEC	ExPEC111	K5	O25:H4	*fimH*30	CTX-M-15	FII_31, FII_36	FIA_4	FIB_1	F31:A4:B1, F36:A4:B1
	ExPEC658	K5	O25:H4	*fimH*30	CTX-M-27	FII_1	FIA_2	FIB_20	F1:A2:B20
	ExPEC761	K5	O25:H4	*fimH*30					
	ExPEC782	Not found	O16:H5	*fimH*41	CTX-M-55	FII_1	FIA_2		F1:A2:B-
	ExPEC904	K5	O16:H5	*fimH*41	CTX-M-14	FII_1	FIA_2	FIB_20	F1:A2:B20
	ExPEC1053	K5	O25:H4	*fimH*30	CTX-M-14		FIA_2		F-:A2:B-
	ExPEC1144	K5	O16:H5	*fimH*41		FII_29		FIB_10	F29:A-:B10
	ExPEC1167	K5	O16:H5	*fimH*41		FII_29		FIB_10	F29:A-:B10
	ExPEC1213	K5	O25:H4	*fimH*30	CTX-M-27	FII_2	FIA_6	FIB_20	F2:A6:B20
	ExPEC1254	K5	O25:H4	*fimH*30	CTX-M-14		FIA_2	FIB_20	F-:A2:B20
	ExPEC1265	K5	O16:H5	*fimH*41		FII_1	FIA_2	FIB_20	F1:A2:B20
	ExPEC1284	K5	O16:H5	*fimH*41	CTX-M-15	FII_1	FIA_1	FIB_16	F1:A1:B16
ST131 of BPEC	ExPEC2220	K5	O25:H4	*fimH*30	CTX-M-27	FII_1	FIA_2	FIB_20	F1:A2:B20
	ExPEC2299	K5	O25:H4	*fimH*30	CTX-M-15	FII_29		FIB_10	F29:A-:B10
	ExPEC2434	K5	O25:H4	*fimH*30	CTX-M-14	FII_35	FIA_2	FIB_20	F35:A2:B20
	ExPEC2459	K5	O16:H5	*fimH*41	CTX-M-27	FII_29		FIB_10	F29:A-:B10
	ExPEC2461	K5	O25:H4	*fimH*30	CTX-M-27	FII_1	FIA_2	FIB_20	F1:A2:B20
	ExPEC2503	K5	O25:H4	*fimH*30	CTX-M-15	FII_35		FIB_10	F35:A-:B10
	ExPEC2583	K5	O16:H5	Not found	CTX-M-27	FII_29, FII_2		FIB_10	F29:A-:B10, F2:A-:B10
	ExPEC2592	K5	O25:H4	*fimH*30	CTX-M-14	FII_1, FII_35	FIA_2	FIB_20	F1:A2:B20, F35:A2:B20
	ExPEC2925	K5	O25:H4	*fimH*30	CTX-M-27	FII_1	FIA_2	FIB_20	F1:A2:B20
	ExPEC2946	K5	O25:H4	*fimH*30	CTX-M-27	FII_1	FIA_2	FIB_20	F1:A2:B20
	ExPEC2960	K5	O25:H4	*fimH*30	CTX-M-15	FII_1	FIA_2	FIB_20	F1:A2:B20
	ExPEC3011	K5	O25:H4	*fimH*30	CTX-M-15	FII_31, FII_36	FIA_4	FIB_1	F31:A4:B1, F36:A4:B1

### Virulence gene profiles and phenotypic validation

Genomic analysis of virulence factors revealed fundamentally different evolutionary strategies between the two clones. ST1193 isolates possessed a highly conserved and stable virulence gene repertoire, with only two isolates harboring additional colibactin genes *(clbA-clbS)*. This core arsenal included several genes, including *vat* (vacuolating autotransporter toxin), *neuABCD* (sialic acid synthesis), *hcp2* (T6SS structural component), *kpsT* (ATP-Binding Cassette Transporter), *espL1* (type III secretion effector), were not found in the ST131 isolates studied here.

**Fig. 3:**
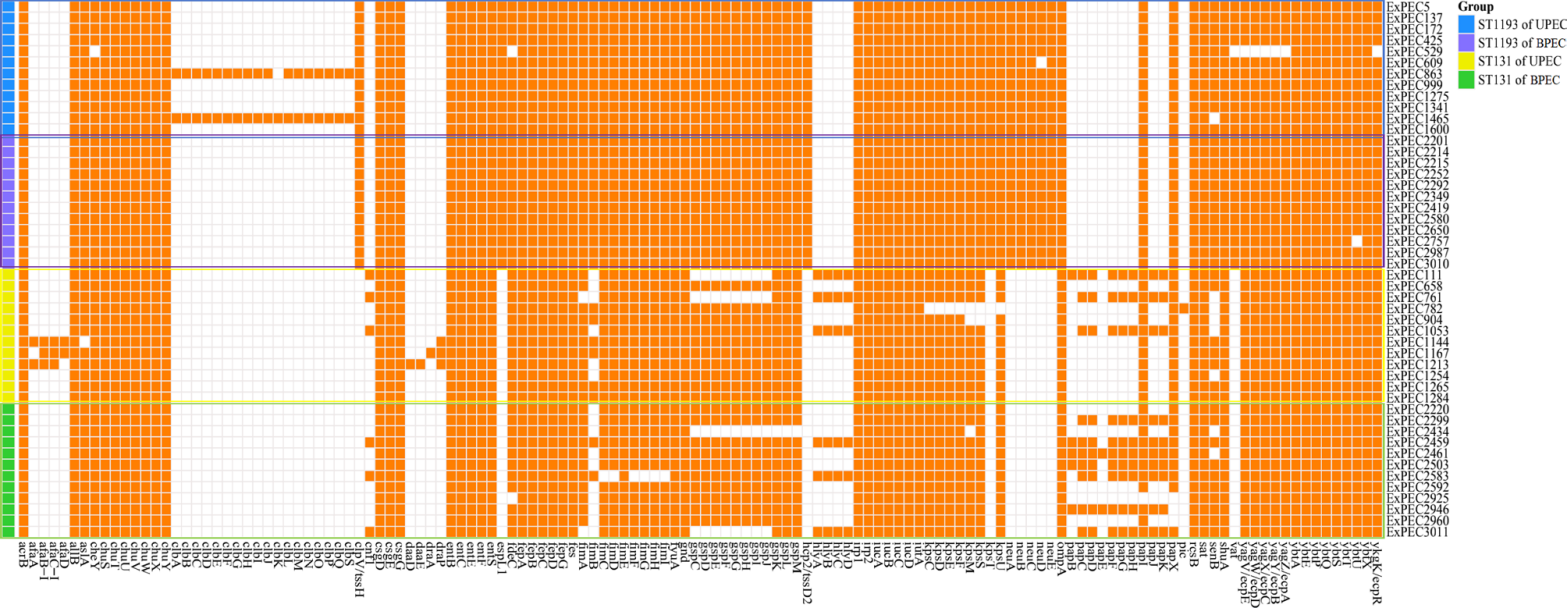
All the virulence genes of the 48 ExPEC isolates

Conversely, ST131 isolates exhibited greater heterogeneity and modularity in their virulence gene content. A subset of six strains carried a complete cytotoxin cluster (*hlyCABD* + *cnf1*), and nearly half had an expanded P fimbriae operon. Some strains carried Afa/Dr adhesin or autotransporter (*pic*) genes. Importantly, partial deletions were observed in core virulence systems (e.g., T2SS, type 1 fimbriae) in some ST131 isolates. The conserved virulence profile of ST1193 may reflect an optimized, generalist strategy, while the modularity of ST131 might allow for more niche-specific adaptation. All the key virulence genes are presented in Fig. 3.

**Fig. 4:**
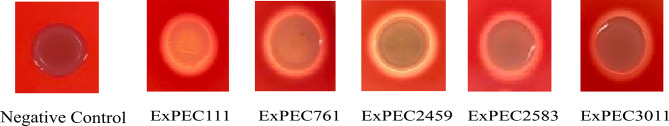
Hemolytic-positive strains

The genotypic predictions were directly validated by phenotypic testing for hemolysin production. As anticipated from the genomic data, hemolytic activity was exclusively observed in the subset of ST131 isolates (5/24) that genotypically possessed the complete *hlyCABD* operon. No hemolysis was detected in any ST1193 isolate or in ST131 isolates lacking the full cytotoxin cluster. This result not only confirms the functionality of the encoded virulence factors but also highlights a clear phenotypic difference in virulence expression between the lineages, underscoring the genotypic distinctions described above. The results of the strains with positive hemolysis test are shown in Fig. 4.

### *G. mellonella* larvae virulence assay

**Fig. 5:**
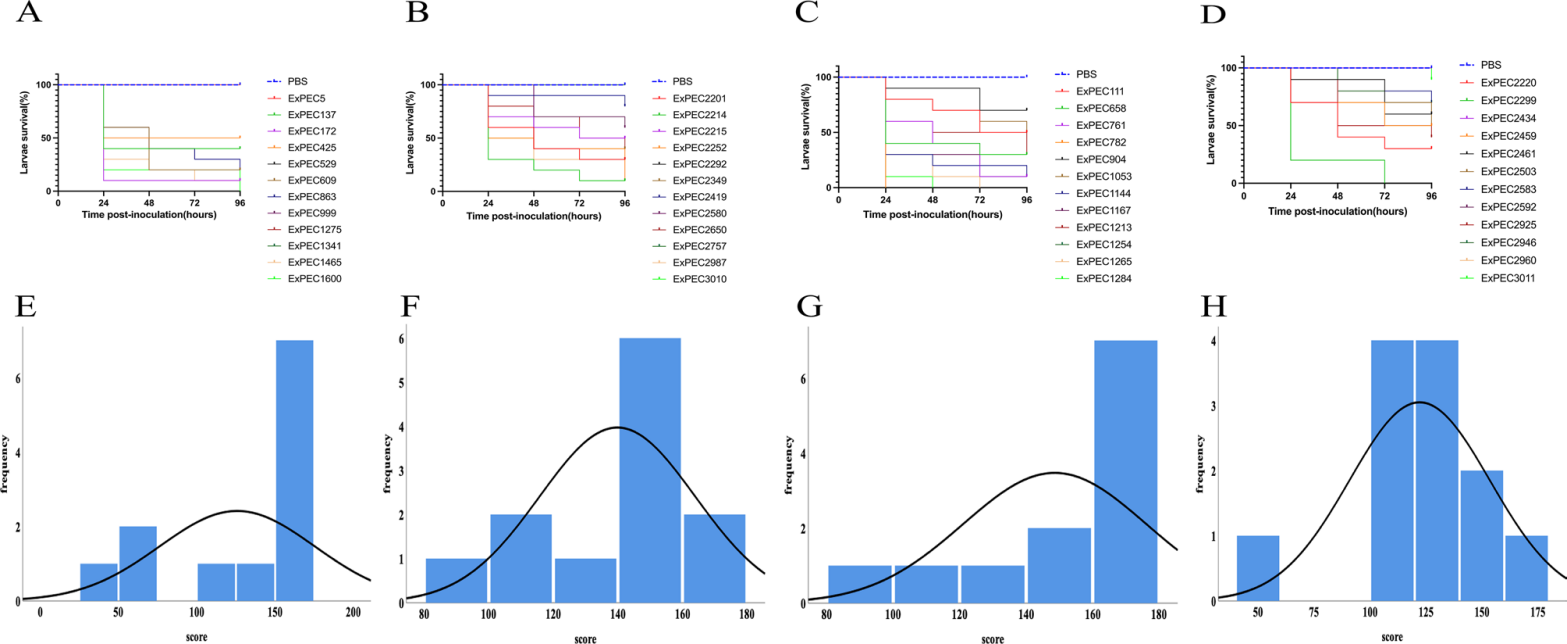
*G. mellonella* larvae survival curves and *G. mellonella* larvae scores of four groups. (**A**-**D**) The *G. mellonella* larvae survival curves of ST1193 group in UPEC, ST1193 group in BPEC, ST131 group in UPEC and ST131 group in BPEC. (**E**-**H**) The *G. mellonella* larvae scores of ST1193 group in UPEC, ST1193 group in BPEC, ST131 group in UPEC and ST131 group in BPEC

The virulence of all 48 isolates was assessed using the *G. mellonella* larvae model. The survival curves and virulence scores for the four groups are presented in Fig. 5. Due to crossing survival curves within each group, formal comparison of survival distributions was precluded. Nevertheless, qualitative differences in curve trajectories were observed across groups. Given the violation of proportional hazards assumptions, we employed the larval scoring system as a quantitative metric for virulence assessment. The Kruskal-Wallis test of virulence scores at 96-hours indicated no statistically significant differences among the four groups overall (*p* = 0.107).

Given the overall null result, we performed exploratory pairwise comparisons. These analyses suggested that virulence was comparable between ST1193 from different sources (UPEC vs. BPEC, *p* = 0.843) and between lineages within the same source (ST1193-UPEC vs. ST131-UPEC: *p* = 0.160; ST1193-BPEC vs. ST131-BPEC: *p* = 0.114). The most notable finding was that ST131-UPEC isolates appeared more virulent than ST131-BPEC isolates (*p* = 0.024) in this model, suggesting a potential source-associated virulence difference within this lineage. For ST1193, the lack of a significant difference between UPEC and BPEC isolates, while not conclusive, could suggest a more consistent virulence phenotype across infection sites that warrants further investigation. The clinical interpretation of these findings requires caution due to the lack of overall statistical significance and the sample size. The results of *G. mellonella* larvae virulence assay was presented in Fig. 5.

## Summary of integrated findings

**Fig. 6:**
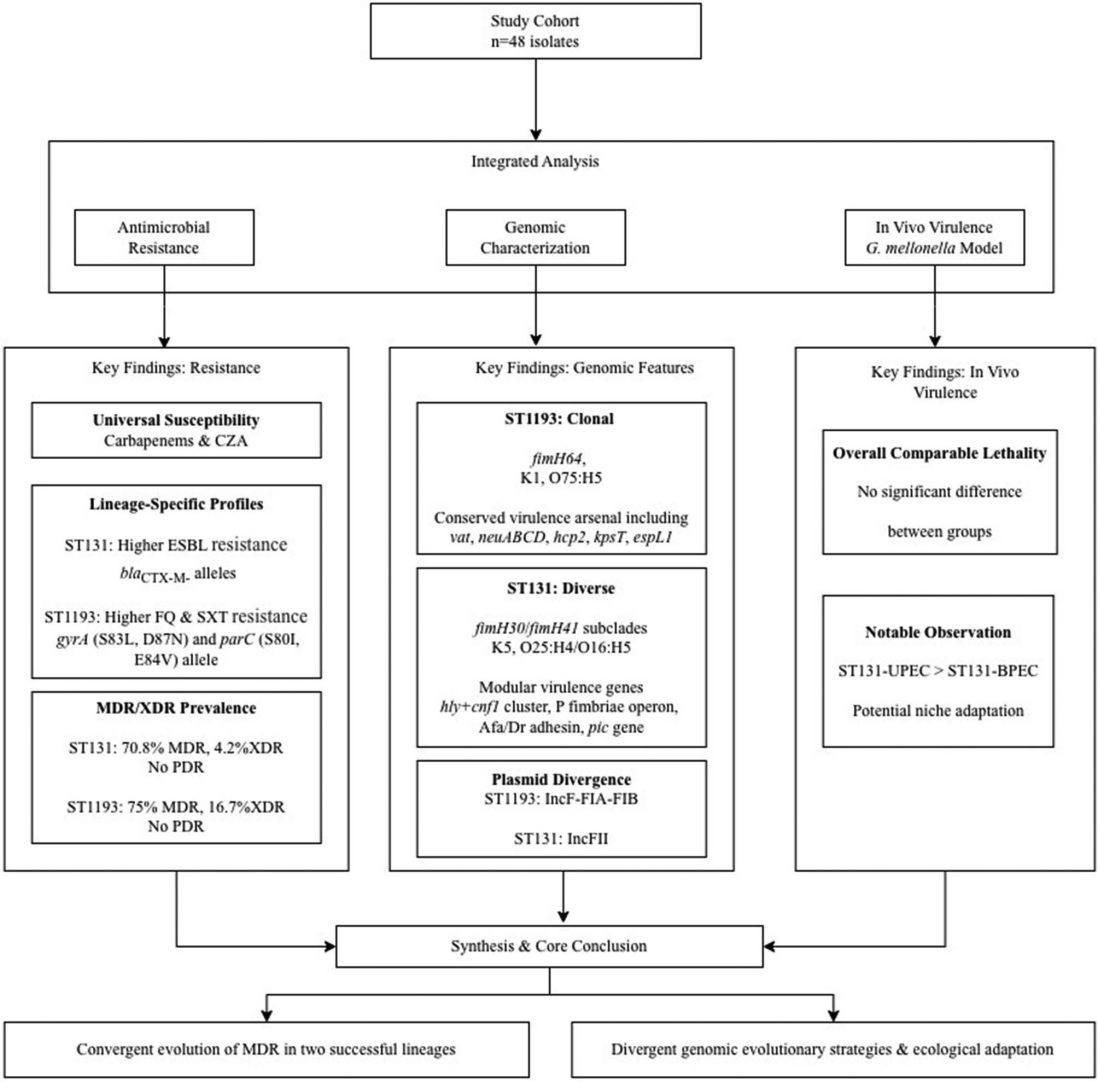
An integrated workflow summarizing the key comparative findings of the study. The diagram illustrates the analytical path from the established cohort through integrated phenotypic and genomic analyses, to the principal findings. The results underscore that ST1193 and ST131 represent two distinct evolutionary paradigms—characterized by clonal stability versus heterogeneous diversity, respectively—while both achieving high-risk epidemic success through convergent multidrug resistance. (CZA: ceftazidime/avibactam; FQ: Fluoroquinolone; SXT: Trimethoprim/sulfamethoxazole; MDR/XDR/PDR: Multidrug-/Extensively drug-/Pan-drug-resistant; UPEC/BPEC: Urinary/Bloodstream infection isolate)

A schematic overview integrating the main findings from the comparative phenotypic and genomic analyses is presented in Fig. 6, which summarizes the study’s core conclusions.

## Discussion

Extra-intestinal pathogenic *Escherichia coli* (ExPEC) represents a major global health concern, responsible for a vast spectrum of infections beyond the intestine, including urinary tract infections, bloodstream infections, and meningitis [47]. Among these, *E. coli* ST131 is a globally dominant epidemic lineage and multidrug-resistant clone, whereas *E. coli* ST1193 represents an emerging fluoroquinolone-resistant clone exhibiting rapid worldwide dissemination [48–51]. The success of such ExPEC clones is inherently linked to their ability to navigate the complex interplay between antimicrobial resistance, virulence, and host-microbiota dynamics [52, 53]. Although extensively studied, ST131’s success mechanisms remain critical to understand [54]. Conversely, knowledge of ST1193 is severely limited despite its alarming prevalence in bloodstream and urinary tract infections. This expanding threat positions ST1193 as a potential successor to ST131 as the next predominant pathogenic *E. coli*. To comprehensively compare these two lineages, we employed WGS—a method chosen for its superior ability over traditional approaches like microarrays to provide an unbiased, hypothesis-free analysis of the entire genetic landscape, including novel mutations and structural variations [55]. This approach was particularly suited for capturing the full genetic repertoire of the emerging ST1193 clone and for enabling high-resolution phylogenetic and functional comparisons with ST131. This study provides a comparative genomic and phenotypic analysis of these two lineages, with a specific focus on isolates from both urinary tract and bloodstream infections, to identify distinguishing features that may contribute to their success.

Our findings on antimicrobial resistance are largely consistent with previous reports which established the strong association of ST131 with ESBL production and of ST1193 with fluoroquinolone resistance [56, 57]. We not only corroborate these classic patterns but also extend them through detailed statistical and genetic analyses. Firstly, the significantly higher ESBL prevalence in ST131 (83.3% vs. 50% in ST1193, *p* < 0.05) underscores a fundamental difference in their primary resistance mechanisms. Secondly, a novel and critical observation from our stratified cohort design was that ESBL producers were significantly enriched in bloodstream isolates within both lineages (ST1193-BSI: 8/12 vs. ST1193-UTI: 4/12; ST131-BSI: 12/12 vs. ST131-UTI: 8/12). This consistent pattern across genetically distinct clones is statistically compelling and strongly suggests that ESBL carriage may confer a selective advantage for systemic invasion. This advantage could stem from enhanced bacterial survival against host defenses in the bloodstream or the frequent use of empirical cephalosporin therapy for suspected BSIs [58]. Thirdly, the universal fluoroquinolone resistance in ST1193 was genetically explained by the characteristic *gyrA* (S83L, D87N) and *parC* (S80I, E84V) mutations, a well-documented hallmark of this clone, demonstrating perfect genotypic-phenotypic concordance [59]. Finally, and from a public health perspective, the application of standardized international definitions revealed a high prevalence of multidrug-resistant (MDR) phenotypes in both lineages (70.8% in ST131, 75% in ST1193), with ST1193 exhibiting a notably higher proportion of extensively drug-resistant (XDR) isolates (16.7% vs. 4.2%). Although this difference in XDR prevalence was not statistically significant with our sample size, the trend is clinically alarming. This trend, coupled with the distinct plasmid replicon profiles (IncF-FIA-FIB dominant in ST1193 vs. IncFII in ST131) that create a genetic backdrop permissive for the acquisition and maintenance of diverse resistance genes [60], indicates that ST1193 may be actively accumulating a broader resistance repertoire, warranting close future surveillance [61].

A central finding of our study is the stark contrast in genomic stability and virulence profiles between the two lineages. Our genomic data statistically confirm that ST1193 exhibited a remarkably conserved and stable genetic backbone, characterized by near-uniformity in its serotype (O75:H5), capsule type (K1), *fimH64* allele, and virulence gene profile. This extreme homogeneity, with 23 out of 24 (95.8%) ST1193 isolates carrying the *fimH64* allele, strongly suggests a recent clonal expansion from a common ancestor. This conserved arsenal includes a set of unique genes implicated in host adaptation: *vat* (vacuolating autotransporter toxin, enhancing sepsis fitness [62]), *hcp2/clpV* (T6SS components, mediating bacterial competition [63, 64]), and *neuABCD*/*kpsT* (enabling K1 capsule biosynthesis and conferring serum resistance [65, 66]). These features collectively point towards a highly optimized and stable genotype, potentially reflecting a “generalist” adaptation strategy. In contrast, ST131 demonstrated significant heterogeneity in these same features, segregating into classic subclones and exhibiting a modular, variable repertoire of virulence genes (e.g., cytotoxin clusters *hlyCABD* + *cnf1* [67–69], P fimbriae operon [70, 71], Afa/Dr adhesins [72], and *pic* autotransporter [73]), alongside genomic evidence of ongoing adaptation, such as partial deletions in core virulence systems like the T2SS operon [74]. This genomic evidence of diversity aligns with its established role as a versatile and adaptable pathogen capable of niche-specific evolution [75].

The ecological implications of these genomic differences were explored using the *G. mellonella* model. It is crucial to frame the interpretation of these results within their statistical context. The Kruskal-Wallis test indicated no statistically significant differences in overall virulence among the four groups (*p* = 0.107). Therefore, our primary and statistically robust conclusion is that the overall virulence potential of ST131 and ST1193, as measured in this model, is comparable, which is consistent with the clinical relevance of both clones [76]. However, exploratory pairwise comparisons suggested an intriguing within-lineage difference: ST131-UPEC isolates appeared more virulent than ST131-BPEC isolates (*p* = 0.024). This observation, while requiring cautious interpretation due to the overall null result and sample size, generates the hypothesis that ST131 may have undergone pathoadaptation to the urinary tract. This notion is supported by its enrichment for urinary virulence factors like Pap fimbriae and aligns with some clinical data suggesting source-associated virulence patterns [77, 78]. For ST1193, the lack of a significant difference between UPEC and BPEC isolates could suggest a more consistent pathogenic potential across infection sites. This would be consistent with its “generalist” genomic profile, a hypothesis that merits further investigation in larger, powered studies.

Although ST1193 and ST131 exhibit comparable levels of drug resistance and virulence—necessitating coordinated preventive measures against both high-risk clones. Our study identifies a critical distinction: their *fimH* adhesion genes encode divergent variants. Consequently, targeting the strain-specific *fimH* signatures presents a promising avenue for developing precision interventions, such as anti-adhesion therapeutics or tailored vaccines, to disrupt colonization mechanisms unique to each clone. The role of FimH warrants careful consideration. While *fimH*64 is a highly conserved marker of ST1193 and *fimH*30 is a defining feature of the ST131-H30 subclade. Our study lacked functional validation, cannot establish these adhesins as deterministic drivers of epidemic success. Their conservation, however, underscores their importance for each clone’s biology. Given the established role of FimH as a key mediator of intestinal colonization and epithelial invasion, this structural divergence likely translates to differential host-pathogen interactions [79, 80]. Therefore, we propose that the FimH adhesin, represents a promising candidate target for future research into novel anti-adhesion therapies or vaccines [81]. This approach could leverage the conserved nature of these alleles within each clone for targeted intervention.

Our study has several important limitations. The sample size, though balanced by design, is moderate, and all isolates originated from a single center, which may limit the generalizability of our findings. The statistical power of the *G. mellonella* virulence assessments were limited, and the intriguing within-ST131 difference requires validation in larger cohorts. Most importantly, the hypotheses generated regarding ecological adaptation and the functional role of specific virulence factors remain speculative and necessitate direct mechanistic validation.

## Conclusion

In conclusion, our integrated analysis reveals that ST1193 and ST131 represent two contrasting evolutionary paradigms achieving convergent epidemic success. The ST131 exemplifies heterogeneity and adaptability, while ST1193 demonstrates remarkable genomic conservation indicative of a recent, successful clonal expansion. Our statistical interpretation uncovered a potential link between ESBL carriage and invasive infection in both clones and highlighted an alarming trend towards broader drug resistance (XDR) in ST1193. The comparable overall virulence in *G. mellonella* underscores the threat posed by both, while exploratory analyses hint at niche-specific adaptation within ST131. Ultimately, the epidemic success of these ExPEC clones appears to be multifactorial, driven by a combination of stable core genomes (including lineage-defining FimH variants) and flexible accessory genomes. The conserved FimH variants emerge as high-priority targets for future research aimed at developing novel, precision interventions, such as anti-adhesion agents, to disrupt the colonization reservoir of these successful clones. Future work should focus on mechanistic validation, multi-center epidemiological studies, and exploring the interplay between these pathogens, host immunity, and the gut microbiota to confirm and expand upon these observations.

## Supplementary Information

Below is the link to the electronic supplementary material.

Supplementary Material 1

Supplementary Material 2

Supplementary Material 3

## Data Availability

The original contributions presented in the study are included in the article. The raw sequencing data for ST131 and ST1193 *E. coli* strains in this study have been uploaded and stored in BioProject PRJNA813378 in the NCBI short read archive. Further inquiries can be directed to the corresponding authors.

## Abbreviations

ExPEC: Extraintestinal pathogenic* E. coli*
UTI: Urinary tract infection
BSI: Bloodstream infection
UPEC: Uropathogenic* E. coli* isolates
BPEC: Bloodstream pathogenic *E. coli* isolates
AMR: Antimicrobial resistance
MDR: Multidrug-resistant
ESBLs: Extended-spectrum β-lactamases
MLST: Multilocus sequence typing
ST: Sequence type
