# Supplementary material for: Comparative genomic and phenotypic analysis of Escherichia coli ST1193 and ST131 from urinary and bloodstream infections: insights into resistance, virulence, and divergent strategies
Source: BMC Infect Dis. 2025 Dec 24;25:1732. doi: 10.1186/s12879-025-12044-5 (PMC12729012; doi:10.1186/s12879-025-12044-5)
Supplement: Supplementary file 1 — Supplementary Material 1 [file 12879_2025_12044_MOESM1_ESM.docx]

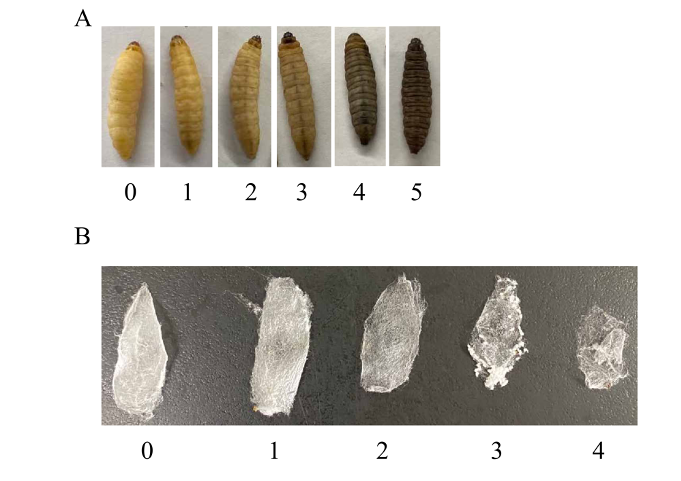


Figure S1. The *G. mellonella* larve scoring system.

(A) Score of the melanization in *G. mellonella* Larve. (B) Score of the cocoon formation in *G. mellonella* Larve.
