# Supplementary material for: Comparative genomic and phenotypic analysis of Escherichia coli ST1193 and ST131 from urinary and bloodstream infections: insights into resistance, virulence, and divergent strategies
Source: BMC Infect Dis. 2025 Dec 24;25:1732. doi: 10.1186/s12879-025-12044-5 (PMC12729012; doi:10.1186/s12879-025-12044-5)
Supplement: Supplementary file 3 — Supplementary Material 3 [file 12879_2025_12044_MOESM3_ESM.docx]

Table S2. The *G. mellonella* Larve Scoring System

| Category | Description | Score |
| --- | --- | --- |
| activity | turn over immediately and move actively | 0 |
|  | turn over quickly and move fast | 1 |
|  | turn over slowly and move slowly | 2 |
|  | longer turning time and move slowly | 3 |
|  | turning time over 10 seconds and minimal movement | 4 |
|  | minimal movement | 5 |
|  | dead | 6 |
| cocoon formation | thick cocoon and completely wrapped | 0 |
|  | thin cocoon and completely wrapped | 1 |
|  | thinner cocoon and completely wrapped | 2 |
|  | thin cocoon silk and partial wrapped | 3 |
|  | a little of cocoon silk | 4 |
|  | no cocoon silk | 5 |
|  | dead | 6 |
| melanization | cream color | 0 |
|  | black tail | 1 |
|  | black back midline and black tail | 2 |
|  | whole body darken | 3 |
|  | whole body darker | 4 |
|  | darkest body or dead | 5 |
